# IL-32γ suppresses lung cancer stem cell growth via inhibition of ITGAV-mediated STAT5 pathway

**DOI:** 10.1038/s41419-019-1737-4

**Published:** 2019-07-01

**Authors:** Yong Sun Lee, Ki Cheon Kim, Raj Kumar Mongre, Ji Young Kim, Yu Ri Kim, Dong Young Choi, Sukgil Song, Jaesuk Yun, Sang-Bae Han, Do Young Yoon, Jin Tae Hong

**Affiliations:** 1 College of Pharmacy and Medical Research Center, Chungbuk National University, Osongsaengmyeong 1-ro, Osong-eup, Heungduk-gu, Cheongju, Chungbuk 28160 Republic of Korea; 2 College of Pharmacy, Yeungnam University, Daehak-Ro 280, Gyeongsan, Gyeongsangbuk Republic of Korea; 3 Department of Bioscience and Biotechnology, Bio/Molecular Informatics Center, Konkuk University, Gwangjin-gu, Seoul 05029 Republic of Korea

**Keywords:** Cancer stem cells, Lung cancer

## Abstract

The cancer stem cells (CSCs) are thought to be responsible for cancer initiation, recurrence, and metastasis via a multifactorial process. IL-32γ has been known to inhibit several tumor developments. However, the role of IL-32γ in CSCs is unknown. The role of IL-32γ on tumor development was assessed in IL-32γ transgenic (Tg) mice allograft and xenograft model. In the in vitro assay, we analyzed CSC growth and apoptosis in cells with IL-32γ overexpression by cell viability assay and tumor-sphere formation assay. In addition, expression of cell proliferation, apoptosis markers, and signaling molecules was determined by western blot analysis. IL-32γ suppressed CD133+ CSC-induced allograft model in IL-32γ Tg mice and xenograft model. Tumor-sphere formation and cell viability assay revealed a greater inhibition of CSC proliferation and antineoplastic activity of IL-32γ in CD133+ CSCs as compared with normal cancer cells. The inhibitory effects of IL-32γ on tumor development were associated with inhibition of the STAT5 pathway. In addition, inhibition of STAT5 increased cleavage of caspase-3, but suppressed CD133 expression and colony formation. Web-based gene network analysis showed that IL-32 is correlated with ITGAV, an integrin gene. Our result revealed that knockdown of ITGAV by siRNA inhibited the phosphorylation of STAT5. Moreover, we identified that ITGAV overexpression reversed the effect of IL-32γ on phosphorylation of STAT5 and the expression of CD133. Our results demonstrate that IL-32γ negatively regulates CD133+ CSC proliferation and tumor development and suggest that IL-32γ has great potential for use in the treatment of cancer progression.

## Introduction

Interleukin-32 (IL-32), a proinflammatory cytokine, was formerly termed as natural killer cells transcript 4 (NK4)^1^. IL-32 is a secreted glycoprotein that exists in nine isoforms and expressed in various tissues, organs, and cell types^2^. IL-32 has nine alternative spliced isoforms, such as IL-32α, IL-32β, IL-32γ, IL-32δ, IL-32ε, IL-32ζ, IL-32η, IL-32θ, and IL-32 small (sm)^3^. Four major isoforms such as IL-32α, IL-32β, IL-32γ, and IL-32δ were initially described from human NK cells^4^. Among these isoforms, IL-32γ is the largest isoform that in the absence of splicing between exon 3 and exon 4, consists of 234 amino acids. IL-32δ lacks the ATG codon located on exon 2, leading to a shift of the ATG codon in exon 3. IL-32α is a spliced form between exon 3 and 4 and exon 7 and 8 of IL-32γ, whereas IL-32β lacks a single splicing region (between exon 3 and 4) found in IL-32γ. Significant roles of IL-32 have been reported in the development of several diseases, such as arthritis, psoriasis, ulcerative colitis, and Crohn’s disease, as well as chronic obstructive pulmonary disease^5,6^. One of the most important isoform IL-32γ is involved in the numerous biophysiological functions, including cancer development^7–9^. The essential phenomenon of IL-32γ-mediated activation of oncogenic switching in cancer-initiating cells is still unclear.

Several reports have demonstrated that cytokines and chemokines have effects on the cancer stem cell niche. Heterogeneous populations of cells, including infiltrating immune cells, stromal cells, and endothelial cells in the cancer stem cell (CSC) niche produce these cytokines and chemokines, including IL-1, IL-6, IL-8, IL-15, and IL-23, which mediate differentiation and proliferation of CSCs^10–13^. In recent literature, it was reported that IL-23 had a significant rise in lung cancer tissues, and regulated lung cancer cell proliferation^14,15^. Moreover, a recent study suggested that IL-32θ inhibits CSC properties in colon cancer^16^.

In lung cancers, one of the key pathways promoting cellular survival or cell growth is Janus kinase/signal transducers and activators of transcription (JAK/STAT) pathway^17,18^. STAT protein family of transcription factors consists of seven members: 1–4, 5A, 5B, and 6^19,20^. Among them, and beside STAT3, the oncogenic activity of STAT5 was documented both in vitro and in vivo. Generally, active STAT5 promotes cell-cycle progression, proliferation, invasion, angiogenesis, and inhibits apoptosis. Constitutive activation of STAT5 is detected frequently in a variety of tumor types^21^. STAT5 signaling has been shown to promote oncogenesis. Moreover, STAT5 contributes to progenitor expansion and differentiation^22^. It has been reported that STAT5 promoting hematopoietic stem cell self-renewal is most crucial in establishment of a leukemic stem cell system.

In the co-expression network data, IL-32 is correlated with integrin alpha V (ITGAV) functioning as a tumor promoter. Integrin gene ITGAV encodes integrin alpha chain V. Increased expression of integrin αVβ3 correlates with disease progression in some human tumors^23,24^. Integrin αVβ3 can also have ligand-independent functions in tumor cells, and recent studies show that unligated integrin αVβ3 can drive cancer cell stemness and drug resistance through activation of K-ras and RalB^25,26^. JAK2/STAT5A is dominantly activated by cell matrix interaction mediated by integrins^27^. However, the role of the integrin-activated STAT5 pathway in CSCs has not been reported yet. In this study, we investigated the molecular mechanisms to elucidate the potential role of IL-32γ in the inhibition of stem-like growth and metastasis by regulation of the ITGAV-mediated STAT5 pathway.

## Results

### Effect of IL-32γ on CSC formation

**Fig. 1:**
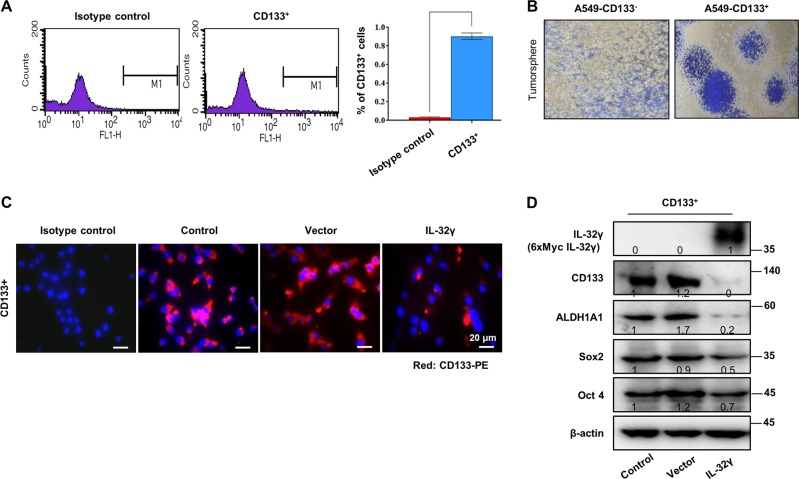
Effect of IL-32γ on CSC formation. **a** Flow-cytometric analysis demonstrates the percentage of tumorigenic CD133+ A549 cells in histogram (CD133-FITC) at the FL1 channel. **b** CD133+/– isolated cells were cultured for 9 days in a six-well plate, which showed self-renewal capacity and tumor-sphere formation efficiency (after sphere formations, cells were stained with Crystal Violet) and photographed. **c** CD133+ A549 CSC cells labeled with the nuclear stain DAPI (blue) and anti-CD133 directly conjugated to PE (red). After staining, cells were observed by fluorescent microscopy for expression and location of CD133. **d** CD133+ A549 CSCs were transfected with IL-32γ or control vector (1 µg/well; six-well plate) for 48 h after seeding, and analyzed by anti-IL-32 (6×Myc-tagged IL-32γ), anti-CD133, anti-ALDH1A1, anti-Sox2, and anti-Oct4 western blotting

Before starting the critical role and mechanisms, we first checked the CSC formation by using A549 NSCLC cell lines. Recently, CD133 is opted as a tumor-initiating CSC marker in most of carcinogenesis. We analyzed the population of CD133+ CSCs from lung adenocarcinoma A549 cells. Flow cytometric analysis of lung cancer cells confirmed the presence of CD133+ cells ( > 1%) in total fraction (Fig. 1a). After 3–4 passages of CD133+/– isolated cell culture, CD133+/– cells were characterized according to their growth pattern. Spheres derived from CD133+ A549 cells readily proliferated and displayed an elongated mesenchymal (stem)-like morphology (Fig. 1b). To investigate the underlying effect of IL-32γ on CSCs, we analyzed the expression of proteins implicated in CSC self-renewal. Notably, CD133 expression is downregulated by IL-32γ in CD133+ A549 CSCs (Fig. 1c, d). Similarly, another CSC biomarkers, aldehyde dehydrogenase 1 family, member A1 (ALDH1A1), Sox2 and Oct4, were significantly reduced in IL-32γ-transfected CD133+ A549 CSCs (Fig. 1d). These results show that IL-32γ plays a key role in the inhibition of CSC progression through it halts the characteristics of cancer stem/progenitor-like cells.

### IL-32γ reduces tumor growth of lung CSC in the allograft model in IL-32γ transgenic mice and in xenograft nude mice model

**Fig. 2:**
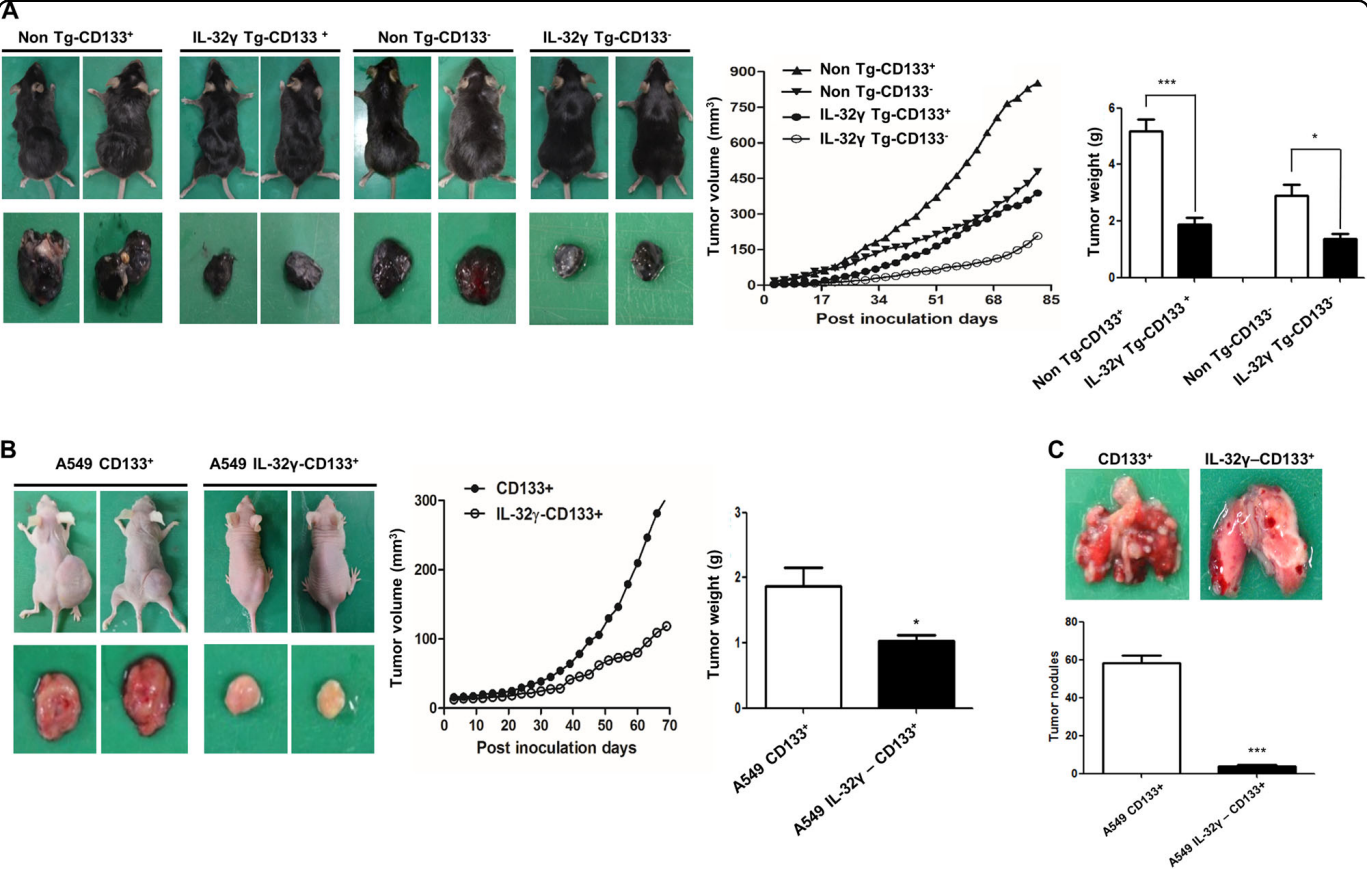
Role of IL-32γ on the tumor growth and tumor metastasis of lung cancer stem cells. **a** Tumor growth inhibition (as assessed by tumor volume) in B16F10 bearing IL-32γ Tg mice. Tumor growth (left panel) from mice injected with CD133- B16F10 cells or CD133+ B16F10 cells is shown. Tumor volume (middle panel) and tumor weight (right panel) are shown in **a**. *n* = 8, *p < 0.05; ***p < 0.001. **b** Overexpression of IL-32γ inhibits the tumor growth of lung cancer stem cells in xenografts. Tumors from mice injected with control vector- or IL-32γ-CD133+ A549 cells are shown. Tumor volume (middle panel) and tumor weight (right panel) are shown in **b**. *n* = 8, *p < 0.05. **c** Overexpression of IL-32γ inhibits the metastasis of lung CSCs in xenografts. Metastasized tumor nodules from mice injected with control vector- or IL-32γ-CD133+ A549 cells are shown. Tumor nodules were measured after the mice were killed. *n* = 6, ***p < 0.001

First, to explore the antitumor effect of IL-32γ in lung CSCs in vivo, we injected CD133+ and CD133- CSCs into non-Tg and IL-32γ Tg mice. As shown in Fig. 2a, tumor growth of the CD133+ CSCs was significantly inhibited in IL-32γ Tg mice compared with non-Tg mice. Next, we also injected the IL-32γ expressing CD133+ CSCs or normal CD133+ CSCs into nude mice to confirm that IL-32γ could suppress tumor growth. In agreeing with the allograft model, we showed that tumor progression was significantly inhibited in the mice inoculated with IL-32γ expressing CD133+ cells as compared with control in the xenograft model (Fig. 2b). To further examine the activation and expression status of pSTAT5, CD133, ALDH1A1, and other CSC regulatory proteins, the mice tumor sections were subjected to immunohistochemistry to study the histology and expression of key proteins being examined in this study. We showed that the expression of CSC markers, such as CD133 and ALDH1A1, and activation of STAT5, were much lower in the tumor of IL-32γ Tg mice as compared with the non-Tg mice (Supplementary Fig. 1A). Moreover, the expression of CDK6, MMP-2, and PCNA and nuclear translocation of p65 were inhibited by IL-32γ (Supplementary Fig. 1B). We also confirmed that the expression of CD133 and nuclear translocation of STAT5 was much greatly inhibited in tumors of nude mice injected with IL-32γ expressing CD133+ CSCs compared with the control (Supplementary Fig. 1C).

### IL-32γ inhibits tumor metastasis in CD133+ induced xenograft nude mice model

To evaluate the effect of IL-32γ on cancer stem cell metastasis, we intravenously injected the IL-32γ expressing CD133+ CSCs into athymic nude mice. We observed that CD133+ CSCs augmented lung metastasis, while IL-32γ-transfected CD133+ CSC-inoculated animals showed significantly less metastasized tumor (Fig. 2c). These results suggest that the induction of IL-32γ by the CD133+ CSC tumor is required for eliciting an effective antitumor efficacy. We further examined the activation and expression status of pSTAT5 and CD133 by immunohistochemistry. In agreement with the status in metastasis, the expression of CD133 and nuclear translocation of pSTAT5, but not STAT1 and STAT3, was decreased in lung tissue injected with IL-32γ expressing CD133+ CSCs (Supplementary Fig. 1D). These results collectively demonstrated that IL-32γ negatively modulates the expression and activation of CD133 and pSTAT5 in vivo and is effective in suppressing tumorigenic potential and metastasis in the IL-32γ transgenic mouse model.

### IL-32γ inhibits growth of CD133+ cancer stem cells through cell-cycle arrest and apoptosis

**Fig. 3:**
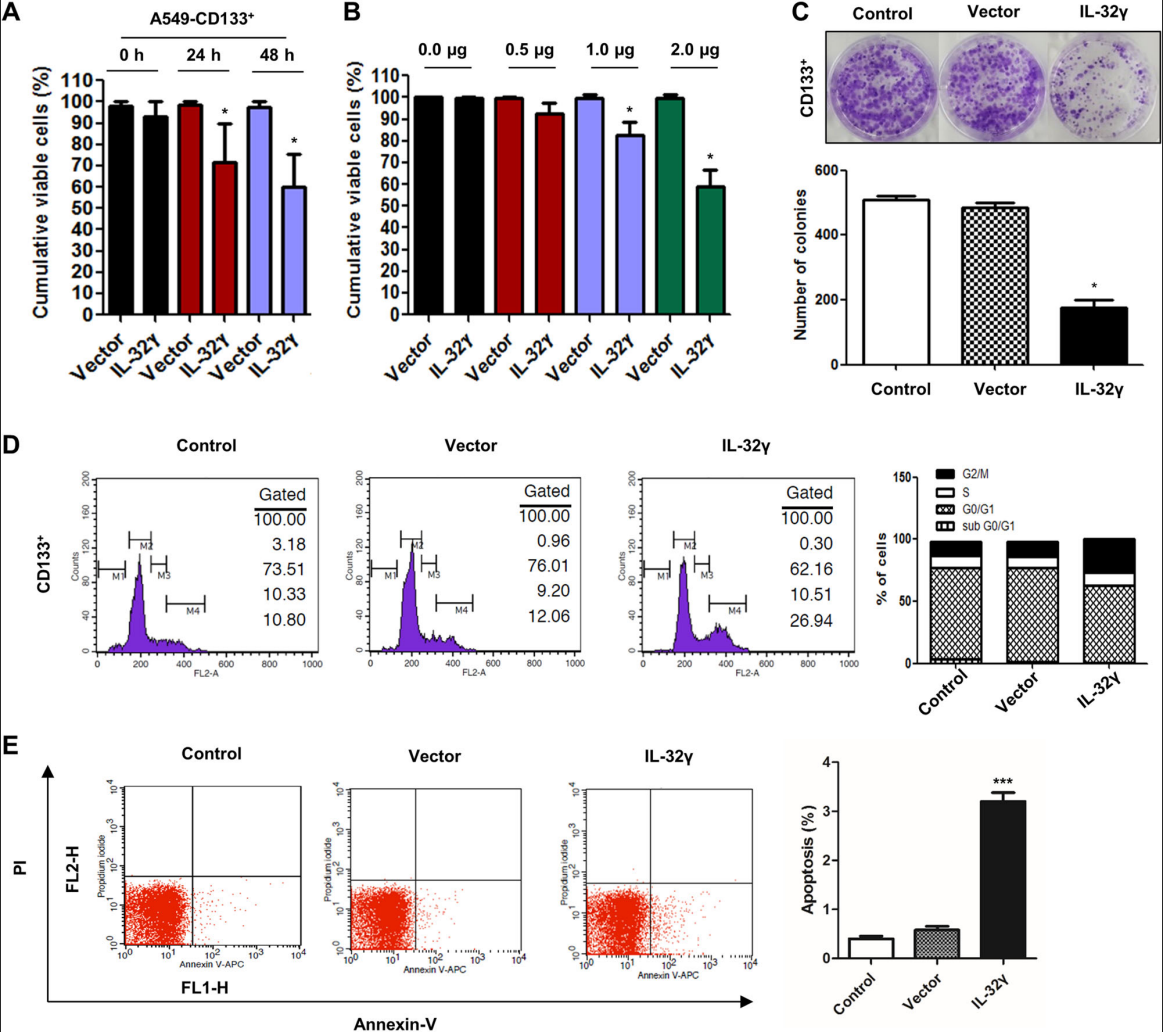
Role of IL-32γ on inhibition of lung cancer stem cells. **a** To study the potential role of IL-32γ against CSCs, CD133+ cells were cultured at 60–70% confluency and transfected with human IL-32γ vector as 1 µg/well of a six-well plate, after 0, 24, or 48 h of post transfection; cell proliferation assay was performed to investigate the efficacy of IL-32γ against CSCs. *p < 0.05. **b** CD133+ cells were transfected with human IL-32γ vector as 0, 5, 1, or 2 µg/well of a six-well plate, after 48 h of post transfection; cell proliferation assay was performed to investigate the efficacy of IL-32γ against CSCs. *p < 0.05. **c** Colony formation by control CD133+ and IL-32γ-CD133+ A549 cells. CD133 + isolated cells were cultured for 9 days in a six-well plate, which showed self-renewal capacity and tumor-sphere formation efficiency (after sphere formations, cells were stained with Crystal Violet) and photographed at 20× resolution. *n* = 5, *p < 0.05. **d** CD133+ cells were transfected with the IL-32γ vector at 2 µg or treated as mock/control for 48 h. Cellular DNA content was determined by flow cytometry, and the relative cell-cycle distribution is given in percentage. Total relative number of arrested cells at G2/M checkpoints have been plotted with a bar graph from three independent experiments. **e** CD133+ cells were transfected with human IL-32γ for 24 h, and then labeled with Annexin-V/PI. After that, apoptosis was analyzed by flow cytometry as described in the “Materials and methods” section. Data are representative of three independent experiments performed in triplicates. ***p < 0.001

To explore the potential role of IL-32γ on cell growth of CD133+ CSCs, CD133+ cells were transfected with IL-32γ and evaluated the cell viability by MTT assay. As shown in Fig. 3a, IL-32γ expressing CD133+ CSCs showed a 20–30% reduced growth as compared with vector-transfected cells. We also found that IL-32γ significantly reduced cell growth in a concentration-dependent manner (Fig. 3b). Colony-forming tumor-sphere cultures revealed a greater inhibition of proliferation by IL-32γ (Fig. 3c). As extensively accepted, cancer stem cells always exert a characteristic uncontrolled growth with various phases of the cell cycle. Many clinically used therapies for cancer treatment were specially targeted to cell-cycle arrest, and this was considered as a promising lead to cancer therapeutics. So, we wondered if IL-32γ contributes to play a tumoricidal role through arrest of cancer stem cells at the G2/M checkpoint of the cell cycle, and the cell-cycle distribution of CD133+ cells after IL-32γ induction was analyzed through FACS assay. We found that IL-32γ-expressing CD133+ CSCs were blocked in the G2/M phase, compared with control and vector-transfected cells (Fig. 3d). We also showed that apoptotic cell death was significantly increased in IL-32γ-transfected CD133+ CSCs compared with control and vector-transfected cells in Annexin-V apoptosis assay (Fig. 3e). These results suggest that overexpression of IL-32γ inhibited lung cancer stem cell growth, possibly by inducing cell-cycle arrest as well as apoptosis.

### IL-32γ plays a crucial role in expression of cell-cycle and apoptosis-related proteins in cancer stem cells

**Fig. 4:**
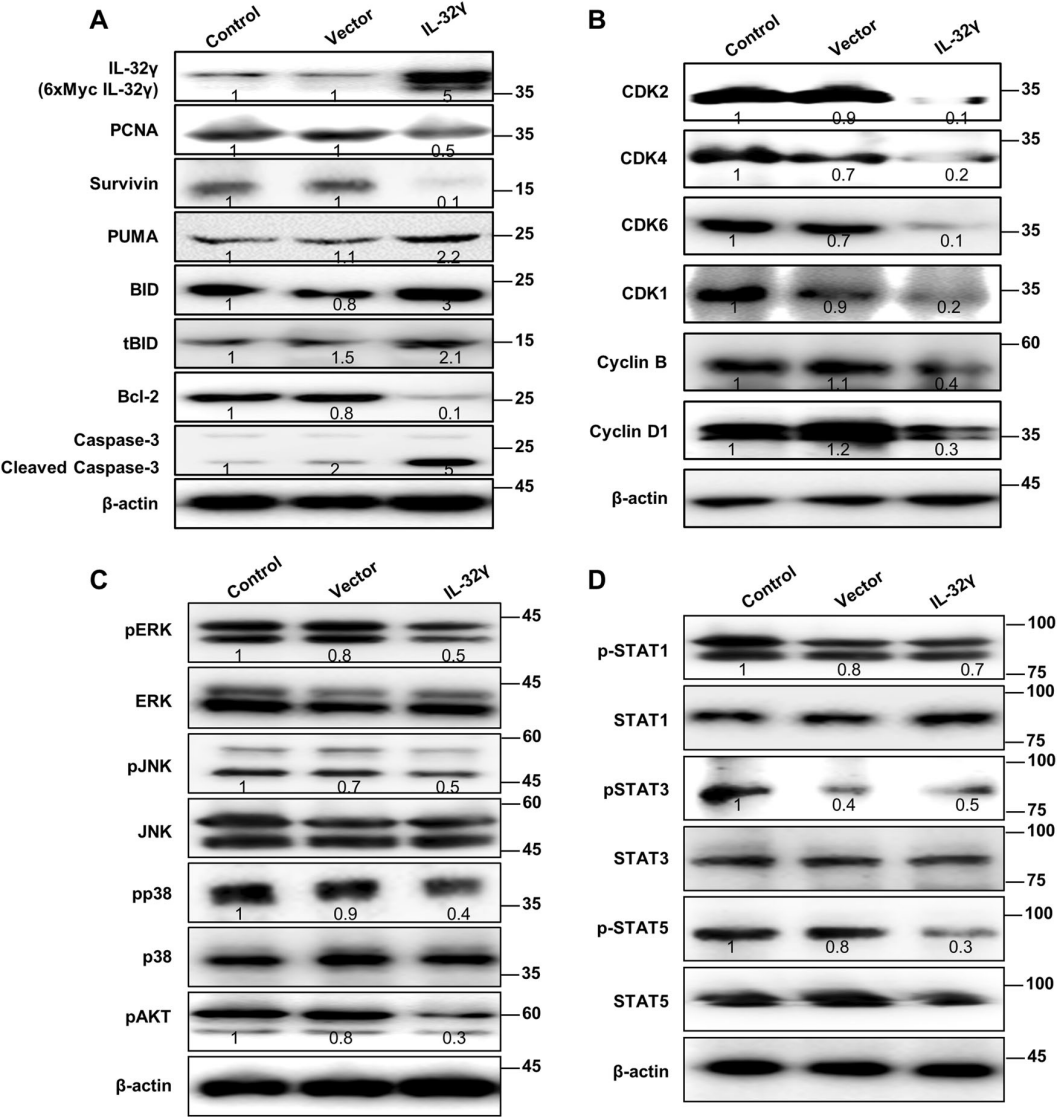
Effect of IL-32γ on the expression of cell-cycle and apoptosis-related proteins in cancer stem cells. **a** CD133+ A549 cells were transfected with human IL-32γ, after 24 h of post transfection; cell survival and apoptosis regulatory proteins, including PCNA, Survivin, BID, tBID, PUMA, Bcl-2, and caspase-3 were detected using specific antibodies. **b** Cell-cycle regulatory proteins, including CDK1, CDK2, CDK4, CDK6, and Cyclin B and D1 were detected using specific antibodies. **c** Expression of MAPK pathway related proteins, including pERK, pJNK, pp38, and pAKT was detected using specific antibodies. **d** Activation of STAT pathway regulatory proteins was detected using specific antibodies, such as pSTAT1, pSTAT3, and pSTAT5. β-actin protein was used as an internal control. Each band is representative of three independent experiments

Next, we then checked the expression of CSC regulatory proteins. We showed that overexpression of IL-32γ in CD133+ cells, significantly inhibited tumor cell growth marker protein survivin compared with control cells (Fig. 4a). The similar inhibitory expression patterns of cancer progression proliferation marker PCNA in CD133+ cells indicate that IL-32γ can suppress the carcinogenic properties of CSCs as compared with control. To confirm the apoptotic effect of IL-32γ, we showed the expression of other apoptotic proteins. We showed that IL-32γ induced expression of BID and tBID, as well as PUMA in CD133+ CSCs. In addition, the inversely relative expression of Bcl-2 has been downregulated after transfection of IL-32γ in CD133+ cells. We found that IL-32γ induction strongly upregulated caspase-3 in CD133+ cells. We already showed the effect of IL-32γ on DNA synthesis by FACS analysis, so we next come to know the possible mechanism action of cell-cycle arrest by the IL-32γ cytokine in CSCs. Interestingly, we found that elevated protein levels of CDK1, CDK2, CDK4, and CDK6 have been dramatically decreased, as compared with control as well as vector. The inhibition of CDK takes places at several levels, including the regulation of Cyclin B and Cyclin D1, and its binding to CDK (Fig. 4b). Next, we also showed the MAPK pathways, because the MAPK pathway plays a prominent role in maintaining the stem-like phenotype of many CD133+ cancer stem cells (Fig. 4c). We found that IL-32γ inhibits phosphorylation of ERK, p38, and AKT. These results suggest that IL-32γ suppresses the CSC proliferation through inhibition of the phosphorylation of ERK, p38, and Akt.

### Inhibition of constitutive activation of STAT5 signaling in CD133+ cancer stem cells by IL-32γ

**Fig. 5:**
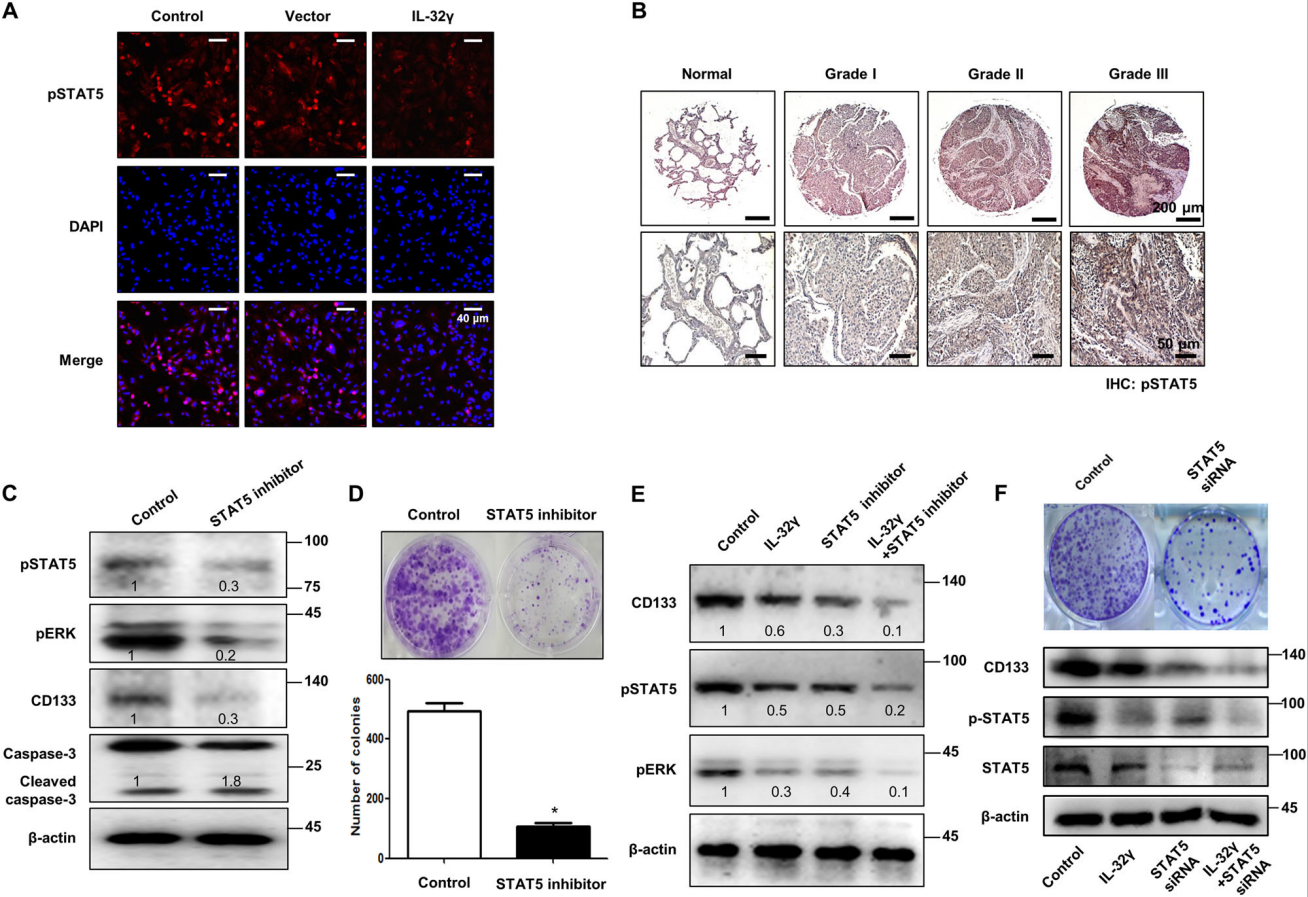
IL-32γ inhibits constitutive activation of STAT5 signaling in CD133+ cancer stem cells. **a** CD133+ cells were transfected with human IL-32γ, after 24 h of post transfection; expression of pSTAT5 was determined by immunofluorescence using a confocal microscope. **b** Human normal lung or NSCLC tissue sections (Grade I–III) were processed and stained; immunohistochemistry analyses for the expression of pSTAT5 were performed. The figures represent the sample of each cancer grade. **c** CD133+ A549 cells were treated with the STAT5 inhibitor (30 μM) for 24 h, and the expression of pSTAT5, pERK, CD133, and cleaved caspase-3 was determined by western blotting**. d** Effect of the STAT5 inhibitor on the colony formation of CD133+ CSCs. CD133+ isolated cells were cultured with or without the STAT5 inhibitor (30 μM) for 9 days in a six-well plate, which showed self-renewal capacity and tumor-sphere formation efficiency (after sphere formations, cells were stained with Crystal Violet) and photographed at 20× resolution. *n* = 5, *p < 0.05. **e** CD133+ A549 cells were transfected with human IL-32γ, after 24 h of post transfection, and it was treated with the STAT5 inhibitor (30 μM) for another 24 h. After that, pSTAT5, pERK, CD133, and cleaved caspase-3 determined by western blotting were detected using specific antibodies. **f** CD133+ A549 cells were transfected with human IL-32γ, after 24 h of post transfection, and it was transfected with STAT5 siRNA (100 nM) for another 24 h. After that, pSTAT5 and CD133 were determined by western blotting. Each band is representative of three independent experiments

The STAT pathways have been found to be constitutively active in various human cancers, including lung cancer, and their activation is believed to play a critical role in the stem cell phenotype. So, we investigated whether STAT pathways are associated with CSC proliferation. Interestingly, we found that phosphorylated levels of STAT5 were significantly suppressed in IL-32γ-expressed CD133+ cells. However, phosphorylated levels of STAT1 and STAT3 were not changed compared with vector and IL-32γ-transfected cells (Fig. 4d). In the immunocytochemical staining, we showed that expression of pSTAT5 was inhibited by IL-32γ (Fig. 5a). We also found that the expression of pSTAT5 was increased in lung cancer patient tissues (Fig. 5b and Supplementary Fig. 2A). Moreover, we found that the STAT5 inhibitor reduced ERK phosphorylation, CD133 expression, as well as STAT5 phosphorylation, but induced a cleaved caspase-3 level (Fig. 5c). Colony-forming tumor-sphere cultures also revealed that the STAT5 inhibitor greatly inhibited proliferation in CD133+ CSCs (Fig. 5d). To investigate the role of IL-32γ in the mitigation of carcinogenesis via downregulation of the STAT5 pathway, we treated the STAT5 inhibitor after transfection of IL-32γ. We showed that the STAT5 inhibitor and IL-32γ have a synergetic effect on the regulation of CD133+ CSCs by western blotting (Fig. 5e). In addition, we also found that STAT5 knockdown by small-interfering RNA (siRNA) inhibited colony formation, expression of CD133 and phosphorylation of STAT5 similar to the effect of the STAT5 inhibitor, and also has a synergistic effect with IL-32γ on CD133 and pSTAT5 expression (Fig. 5f). These data indicated the significant role of the STAT5 pathway in the inhibitory effect of IL-32γ on the CSC growth.

### Inhibitory effect of IL-32γ on ITGAV signaling in CD133+ cancer stem cells

**Fig. 6:**
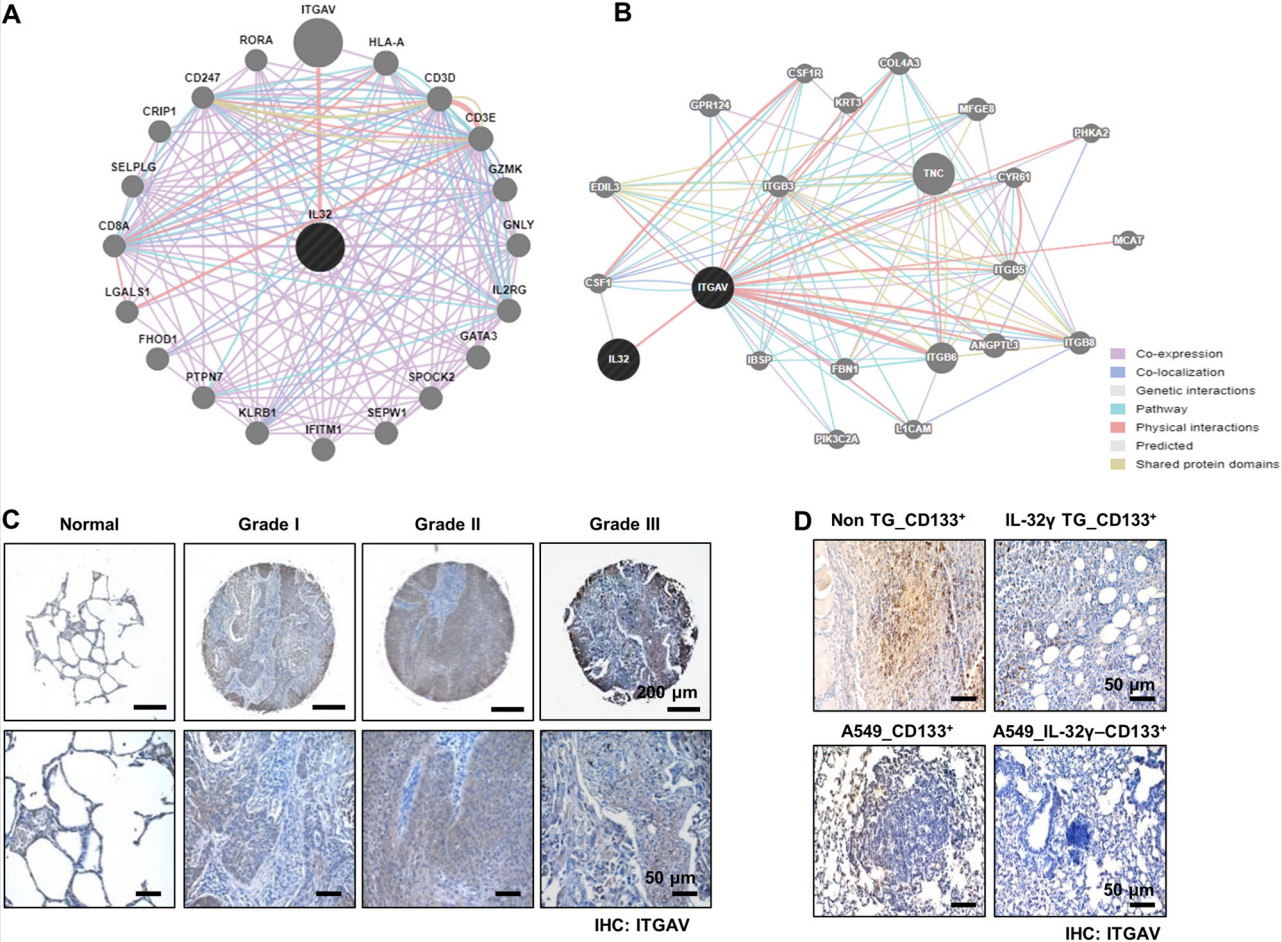
Effect of IL-32γ on the expression of ITGAV. **a**, **b** Gene network analysis using GeneMANIA. ITGAV is the key factor among 20 genes that are related with IL-32 (**a**). The relationships between IL-32 and ITGAV are shown based on known functional association networks (**b**). **c** Human normal lung or NSCLC tissue sections (Grade I–III) were processed and stained; immunohistochemistry analyses for the expression of ITGAV were performed. The figures represent the sample of each cancer grade. **d** Tumors from mice injected with CD133+ B16F10 cells, and then tumors were collected after 85 days. Tumor tissue sections were analyzed by immunohistochemistry for the detection of ITGAV in non-Tg and IL-32γ Tg mice (upper lane). Tumors from nude mice injected with control vector-CD133+ or IL-32γ-CD133+ A549 cells were collected after 70 days. Tumor tissue sections were analyzed by immunohistochemistry for the detection of ITGAV (lower lane)

**Fig. 7:**
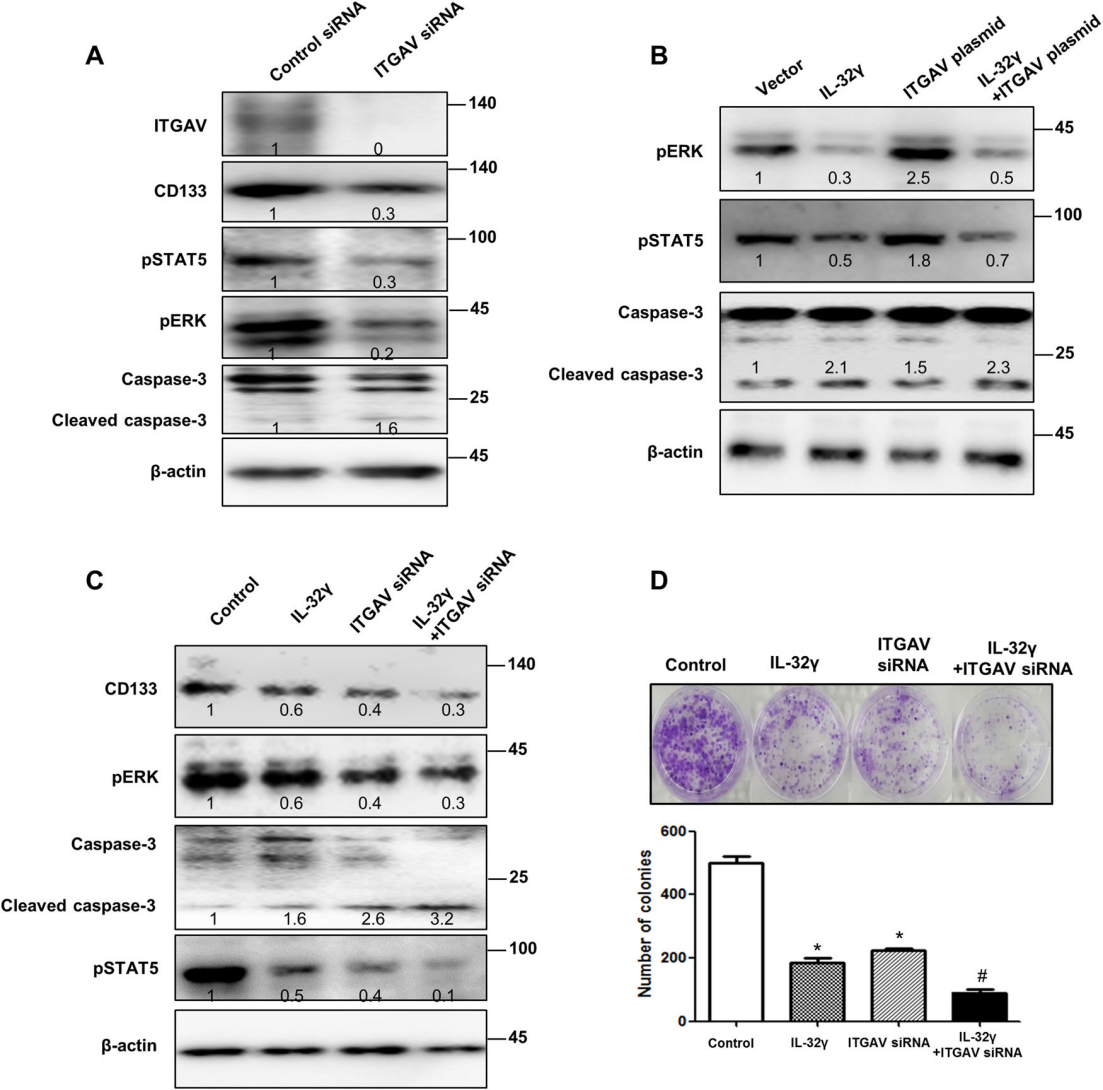
Effect of IL-32γ on ITGAV-mediated STAT5 pathway. **a** CD133+ A549 cells were transfected with ITGAV siRNA (100 nM) for 24 h and then the expression of pSTAT5, pERK, CD133, and cleaved caspase-3 was determined by western blotting. **b** CD133+ A549 cells were transfected with IL-32γ for 24 h and then transfected with ITGAV plasmid for another 24 h. After that, the expression of pERK, pSTAT5, and cleaved caspase-3 was determined by western blotting. **c** CD133+ A549 cells were transfected with IL-32γ for 24 h and then transfected with ITGAV siRNA (100 nM) for another 24 h. After that, the expression of pSTAT5, pERK, CD133, and cleaved caspase-3 was determined by western blotting**. d** CD133+ A549 cells were transfected with IL-32γ for 24 h, and then transfected with ITGAV siRNA (100 nM) for another 24 h and cultured for 9 days in a six-well plate, which showed self-renewal capacity and tumor-sphere formation efficiency (after sphere formations, cells were stained with Crystal Violet), and photographed at 20× resolution. *n* = 5, *p < 0.05 (control vs. IL-32γ or ITGAV siRNA); ^#^*p* < 0.05 (ITGAV siRNA vs. IL-32γ + ITGAV siRNA)

Using the web-based gene network analysis, IL-32 is correlated with ITGAV (Fig. 6a, b). Integrins, such as αvβ3, αvβ5, and α5β1, play a critical role in tumor angiogenesis and tumor growth^27^. In our data, the expression of ITGAV was increased in lung cancer patient tissues (Fig. 6c and Supplementary Fig. 2B). We also found that the expression of ITGAV was decreased in CD133+ CSC-inoculated IL-32γ-Tg mice compared with non-Tg mice (Fig. 6d, upper lane), and in IL-32γ-expressing CD133+ CSC- inoculated nude mice (Fig. 6d, lower lane). Among several integrin-mediated signaling, JAK2/STAT5A is dominantly activated by the cell matrix interaction mediated by integrins. So, we investigated whether IL-32γ inhibits the ITGAV-mediated STAT5 pathway. Our result revealed that knockdown of ITGAV by siRNA inhibited the expression of CD133, pSTAT5, and pERK, but increased the expression of cleaved caspase-3 (Fig. 7a). We showed that ITGAV overexpression reversed the effect of IL-32γ on phosphorylation of STAT5, and the expression of CD133 as well as cleaved caspase-3 (Fig. 7b). We also showed that ITGAV siRNA and IL-32γ have a synergetic effect on the regulation of CD133+ CSCs by western blotting with CD133, pSTAT5, pERK, and cleaved caspase-3 (Fig. 7c). Moreover, colony-forming tumor-sphere cultures revealed that ITGAV siRNA and IL-32γ greatly inhibit the proliferation of CD133+ CSCs (Fig. 7d). We additionally found that treatment of siRNA of IL-32 increased p-STAT5 and ITGAV expressions and colony formation, but decreased cleaved caspase-3 expression (Supplementary Fig. 3). These results suggest that IL-32γ inhibits the tumorigenic effects of CD133+ CSC through inhibition of ITGAV-regulated STAT5 pathways.

## Discussion

IL-32 and its isoforms are correlated in various diseases. Its aberrant production is linked to oncogenesis, the progression of multiple types of cancer, and the suppression of tumors^7,9,28,29^. Among them, IL-32γ is the functional active form. There are several studies suggesting that IL-32γ has a critical role for tumor-suppressive effect. As an example, IL-32γ enhances TNF-α-induced cell death in cases of colon cancer and inhibits the growth of cancer cells by blocking the NF-κB and STAT3 pathways^7,9^. Besides the anticancer effect of IL-32γ, a recent study suggested that IL-32Ɵ reduced the self-renewal activity and the stem cell niche of colon cancer stem cells. However, the role of IL-32γ on the oncogenic switching in cancer-initiating cells and cancer stem cell niche, as well as the critical mechanisms, has not been reported yet. So, in this study, we investigated the molecular mechanisms to elucidate the potential role of IL-32γ on the inhibition of stem-like growth in vivo and in vitro.

To identify the antitumor effect of IL-32γ in lung cancer stem cells in vivo, we injected CD133+ and CD133- CSCs into non-Tg and IL-32γ Tg mice. We observed that tumor growth of the CD133+ CSCs was inhibited in IL-32γ Tg mice compared with non-Tg mice. In agreeing with the allograft model, we showed that tumor progression was significantly inhibited in the mice inoculated with IL-32γ-expressing CD133+ cells as compared with control. Moreover, we also observed that CD133+ CSCs augmented lung metastasis, while IL-32γ-transfected CD133+ CSC-inoculated animals showed significantly less metastasized tumor. From these results, we suggest that the IL-32γ is also required for eliciting an effective antitumor efficacy in CD133+ CSC-induced lung tumor development.

In the web-based gene network analysis data, IL-32 is mainly correlated with ITGAV. ITGAV is known to be associated with tumor development. ITGAV expression was elevated in the colon cancer patient tissues^30^. Increased expression of integrin αVβ3 correlates with a poor prognosis in some human tumors^23^. Integrin αVβ3 can drive cancer cell stemness and drug resistance^31^. Surprisingly, JAK2/STAT5A is dominantly activated by cell matrix interaction mediated by integrins^27^. However, the role of the integrin-activated STAT5 pathway in CSCs has not been reported yet. So, we demonstrated the molecular mechanisms to elucidate the potential role of IL-32γ in the inhibition of stem-like growth and metastasis by regulation of ITGAV. In our study, the expression status of pSTAT5 and ITGAV was dramatically decreased by introduction of IL-32γ, in agreeing with the decreased expression of CD133 in tumor sections and CSCs. In addition, knockdown of ITGAV inhibits the expression of CD133, pSTAT5, and pERK, but increased the expression of cleaved caspase-3. We further found that ITGAV overexpression reversed the effect of IL-32γ on the expression of CD133. We also showed that ITGAV siRNA and IL-32γ have a synergetic effect on the regulation of CD133+ CSCs by western blotting with CD133, pSTAT5, pERK, and cleaved caspase-3. These data suggest that suppression of the ITGAV signal could be significant for IL-32γ-induced CSC-oriented lung tumor development.

CSCs always exert a characteristic uncontrolled growth with various phases of the cell cycle. Many clinically used therapies for cancer treatment were specially targeted to cell-cycle arrest, and this was considered as a promising lead to cancer therapeutics. As expected, our result revealed that IL-32γ-expressing CD133+ CSCs were blocked in the G2/M phase, compared with control. We also showed that apoptotic cell death was significantly increased in IL-32γ-transfected CD133+ CSCs compared with control, suggesting that overexpression of IL-32γ inhibited lung cancer stem cell growth, possibly by inducing cell-cycle arrest as well as apoptosis. Moreover, overexpression of IL-32γ in CD133+ cells, significantly inhibited tumor cell growth marker protein survivin, a member of the inhibitor of apoptosis protein (IAP) family. Further, we showed that IL-32γ induced an apoptotic inducer TRAIL through activation of BID in CD133+ cells as compared with CD133-cells. These apoptotic effects of IL-32γ were also abolished with either by the STAT5 inhibitor or ITGAV siRNA. Moreover, the STAT5 inhibitor suppressed the expression of cleaved caspase-3 and CD133, and colony formation. In addition, we also found that STAT5 siRNA inhibited colony formation and expression of CD133 and phosphorylated STAT5 similar to the effect of the STAT5 inhibitor, and also had a synergistic effect with IL-32γ on CD133 and p-STAT5 expression. It was also found that ITGAV overexpression reversed the effect of IL-32γ on phosphorylation of STAT5. Furthermore, we additionally found that treatment of siRNA of IL-32 increased p-STAT5 and ITGAV expression and colony formation, but decreased cleaved caspase-3 expression. These results suggest that IL-32γ inhibits tumorigenic effects of CD133+ CSC through inhibition of ITGAV-regulated STAT5 pathways.

Cytokines such as IL-1, IL-6, IL-8, IL-15, and IL-23 could regulate apoptosis, differentiation and proliferation of CSCs by control of autocrine and/or paracrine signals to balance between self-renewal and differentiation of CSCs^10–13^. It is also noteworthy that many cytokines, such as IL-15 and thrombopoietin, control CSC growth and expansion through downregulation of the STAT5 pathway^32^. We also found that IL-32θ inhibited the STAT3 pathway-mediated colon cancer stemness^16^. These data suggest that suppression of the ITGAV-mediated STAT5 signal could be significant for IL-32γ-induced CSC-oriented lung tumor development.

In conclusion, our results revealed that a potential isoform of IL-32, IL-32γ inhibits CSC self-renewal and stem cell niche, which is relevant to the growth of tumors and the recurrence of lung cancer through downregulation of the ITGAV-mediated STAT5 pathway.

## Materials and methods

### Reagents and cell culture

A549 lung cancer cells and B16F10 mouse melanoma cells were obtained from the American Type Culture Collection (Manassas, VA, USA). A549 cells and B16F10 were grown at 37 °C in 5% CO_2_-humidified air in Roswell Park Memorial Institute (RPMI) 1640 and Dulbecco Modified Eagle Medium (DMEM) medium that contained 10% fetal bovine serum (FBS), 100 U/ml penicillin, and 100 mg/ml streptomycin/penicillin. CD133+ cells were isolated by CD133 microbead isolation kit (Miltenyi Biotec, Auburn, CA, USA). CD133+/– A549 and B16F10 cells were grown at the same conditions in RPMI 1640 and DMEM medium. RPMI 1640, DMEM, penicillin, streptomycin, and FBS were purchased from Gibco Life Technologies (Grand Island, NY, USA). Negative control siRNA was purchased from Bioneer (Daejeon, Korea) and ITGAV, IL-32, and STAT5 siRNA were purchased from Santa Cruz Biotechnology (Santa Cruz, CA, USA). STAT5 inhibitor (N′-((4-Oxo-4H-chromen-3-yl)methylene)nicotinohydrazide) was purchased from Santa Cruz Biotechnology (Santa Cruz, CA, USA).

### Transfection

Cells were transiently transfected with control vector (pcDNA3.1( + )–6×Myc), IL-32γ vector, or ITGAV vector per well, using WelFect-EX PLUS transfection reagent in OPTI-MEM, according to the manufacturer’s specification (WelGENE, Seoul, Korea)^7^. To generate stable cell lines (control vector and IL-32γ-CD133+ A549 cells), transfected CD133+ A549 cells were cultured in G418 containing growth medium (600 μg/ml) for 4 weeks. G418-resistant colonies were expanded and used.

### Cell viability

Cell viability assay was performed as described previously^33^. Cell viability was determined using 3-(4,5-dimethylthiazol-2-yl)-2,5-diphenyltetrazolium bromide (MTT) solution (5 mg/ml).

### Sphere-formation assay

CD133+/– isolated cells (1 × 10^4^ cells) in culture medium were mixed with 0.35% top agarose and plated onto a six-well plate. The culture medium was changed every 3 days. After 9 days, colonies were stained with crystal violet. Agarose (low-melting) was purchased from Sigma-Aldrich (St. Louis, MO, USA).

### Flow-cytometric analysis

Single-cell-suspended A549 cells were stained with the CD133-FITC antibody (eBioscience, Thermo Fisher, Waltham, MA, USA) for 20 min and washed with FBS-contained Stain buffer (BD Biosciences, San Jose, CA, USA). Stained cells were analyzed by FACS Calibur (BD Biosciences, San Jose, CA, USA).

### Cell-cycle analysis

To examine the cell-cycle distribution of asynchronous populations of lung cancer cells, replicative DNA synthesis and DNA content were analyzed using bivariate flow-cytometric analysis. Control, vector, and IL-32γ-transfected CD133 + A549 CSCs were harvested and fixed in ice-cold 70% ethanol. At least 1–2 h before flow-cytometric analysis, cells were resuspended in a 1-ml aliquot of modified Vindelov’s DNA staining solution (10 μg/ml RNase A and 5 μg/ml propidium iodide in Phosphate Buffered Saline (PBS)). Flow cytometric analysis was done with a flow-cytometry system (FACS Calibur System, BD Bioscience, San Jose, CA, USA). Cells in the G1, S, and G2–M phases of the cell cycle were determined with Modfit LT (Verity House Software, Top-sham, ME, USA).

### In vivo antitumor activity of IL-32γ in the allograft model

Generation of IL-32γ transgenic mice was previously described^7^. In brief, a 705-base-pair fragment of the hIL-32γ gene was subcloned into the EcoRI sites of the pCAGGS expression vector. IL-32γ insertion was confirmed by amplification of genomic DNA isolated from the transgenic mice tails. IL-32γ transgenic (Tg) mice have no overt phenotype compared with WT mice. Male, 6- to 8-week-old IL-32γ Tg and non-transgenic (non-Tg) mice were maintained in accordance with the guidelines prescribed by the Chungbuk National University Animal Care Committee (Cheongju, Korea). Animal experiments were approved and carried out according to the Guide for the Care and Use of Animals (CBNUA-929-16-01, Chungbuk National University Animal Care Committee). CD133 + /– B16F10 cells were injected subcutaneously (5 × 10^5^ cells in 0.1 ml PBS per animal) into Tg mice and non-Tg mice. The tumor volume was monitored twice weekly for 85 days. The tumor volumes were measured with Vernier calipers and calculated using the following formula: (*A* × *B*2)/2, where *A* is the larger and *B* is the smaller of the two dimensions. At the end of the experiment, the animals were killed, and the tumors were separated from the surrounding muscles and weighed.

### In vivo antitumor activity of IL-32γ in a xenograft animal model

Six-week-old male BALB/c athymic mice were purchased from Japan SLC (Hamamatsu, Japan). Control or IL-32γ-expressed CD133 + A549 stable cells were injected subcutaneously (1 × 10^7^ cells in 0.1 ml PBS per animal) into the right-lower flanks of the carrier mice. The tumor volume was monitored twice weekly for 70 days. The formula described above was used to calculate tumor volume. For metastasis assay, cells were intravenously (2 × 10^6^ cells in 0.1 ml PBS per animal) injected into 6-week-old male BALB/c athymic mice, and lung metastasis was assessed after 8 weeks. At the end of the experiment, the animals were killed by cervical dislocation. The tumors were separated from the surrounding muscles and dermis, excised, and weighed.

### Immunohistochemistry

All specimens were formalin-fixed and paraffin-embedded. Hematoxylin and eosin (H&E) and immunohistochemistry staining were performed as described previously^33^. Human tissue microarray slides were purchased from US Biomax (Derwood, MD, USA). Immunohistochemical images were scored by the intensity of staining (0—non-staining, 1—weak staining, 2—moderate staining, and 3—strong staining). Specific antibodies were purchased from Santa Cruz Biotechnology (PCNA, CDK6, pSTAT3, and pSTAT5; Santa Cruz, CA, USA), Abcam (MMP-2, ITGAV, and p65; Cambridge, MA, USA), and Novus Biologicals (CD133 and ALDH1A1; Littleton, CO, USA).

### Immunofluorescence staining

Immunofluorescence staining were done as previously described^33^. CD133 was purchased from Novus Biologicals (Littleton, CO, USA). pSTAT5 was obtained from Santa Cruz Biotechnology (Santa Cruz, CA, USA).

### Western blotting

Western blot analysis was performed as described previously^7^. The membranes were immunoblotted with the specific primary antibodies: PCNA, Bcl-2, pERK, ERK, pJNK, JNK, pp38, p38, pAKT, CDK1, CDK2, CDK4, CDK6, Cyclin B, Cyclin D1, pSTAT1, STAT1, pSTAT3, STAT3, pSTAT5, STAT5, and β-actin (Santa Cruz Biotechnology, Santa Cruz, CA, USA); ITGAV (Abcam, Cambridge, MA, USA); CD133 and ALDH1A1 (Novus Biologicals, Littleton, CO, USA); Survivin, BID, PUMA, and Caspase-3 (Cell Signaling Technology, Beverly, MA, USA). The monoclonal anti-hIL-32 antibody KU32–52 was used as reported previously^7^. Western blot was quantified by ImageJ software.

### Gene network analysis

The gene network of IL-32 was analyzed using the web-based analysis tool GeneMANIA (www.genemania.org), based on the publicly available biological data sets (gene–gene interactions based on attributions: co-expression, co-localization, genetic interactions, pathway, physical interactions, predicted interactions, and shared protein domains).

### Data analysis

The data were analyzed using the GraphPad Prism 4 version 4.03 software (GraphPad Software, La Jolla, CA). Data are presented as means ± S.D. The differences in all data were assessed by one-way analysis of variance (ANOVA). When the *p*-value in the ANOVA test indicated statistical significance, the differences were assessed by the Dunnett’s test.

## Supplementary information

Supplementary figure legend

Supplementary figure 1

Supplementary figure 2

Supplementary figure 3
