# Supplementary material for: IL-32γ suppresses lung cancer stem cell growth via inhibition of ITGAV-mediated STAT5 pathway
Source: Cell Death Dis. 2019 Jul 1;10(7):506. doi: 10.1038/s41419-019-1737-4 (PMC6602938; doi:10.1038/s41419-019-1737-4)
Supplement: Supplementary file 1 — Supplementary figure legend [file 41419_2019_1737_MOESM1_ESM.docx]

**Supplementary Figure 1.** **A-B**, Tumors from mice injected with CD133+ B16F10 cells, and then tumors were collected after 85 days. Tumor tissue sections were analyzed by immunohistochemistry for detection of CD133, ALDH1A1 and pSTAT5 **(A),** and CDK6, MMP-2, p65 and PCNA **(B)** in non-Tg and IL-32γ Tg mice. **C**, Tumors from nude mice injected with A549 CD133+ or A549 IL-32γ-CD133+ cells were collected after 70 days. Tumor tissue sections were analyzed by immunohistochemistry for detection of ALDH1A1, CD133 and pSTAT5. **D**, Metastasized lung tissue from athymic nude mice injected with A549 IL-32γ-CD133+ cells were collected, and tumor tissue sections were analyzed by immunohistochemistry for detection of ALDH1A1, CD133, pSTAT3 and pSTAT5.

**Supplementary Figure 2.** **A-B**, Tissue microarray analysis for expression of pSTAT5 and ITGAV during lung tumor progression in normal, grade Ⅰ, grade Ⅱ and grade Ⅲ tissue samples. Bar graphs showing immunohistochemical (IHC) score of pSTAT5 and ITGAV.

**Supplementary Figure 3.** CD133^+^ A549 cells were transfected with IL32 siRNA (100 nM) for 24 hr and then the expression of pSTAT5, STAT5, ITGAV and cleaved caspase-3 was determined by Western blotting**.** Each band is representative of three independent experiments.
