# Supplementary figures and images for: IL-32γ suppresses lung cancer stem cell growth via inhibition of ITGAV-mediated STAT5 pathway
Source: Cell Death Dis. 2019 Jul 1;10(7):506. doi: 10.1038/s41419-019-1737-4 (PMC6602938; doi:10.1038/s41419-019-1737-4)

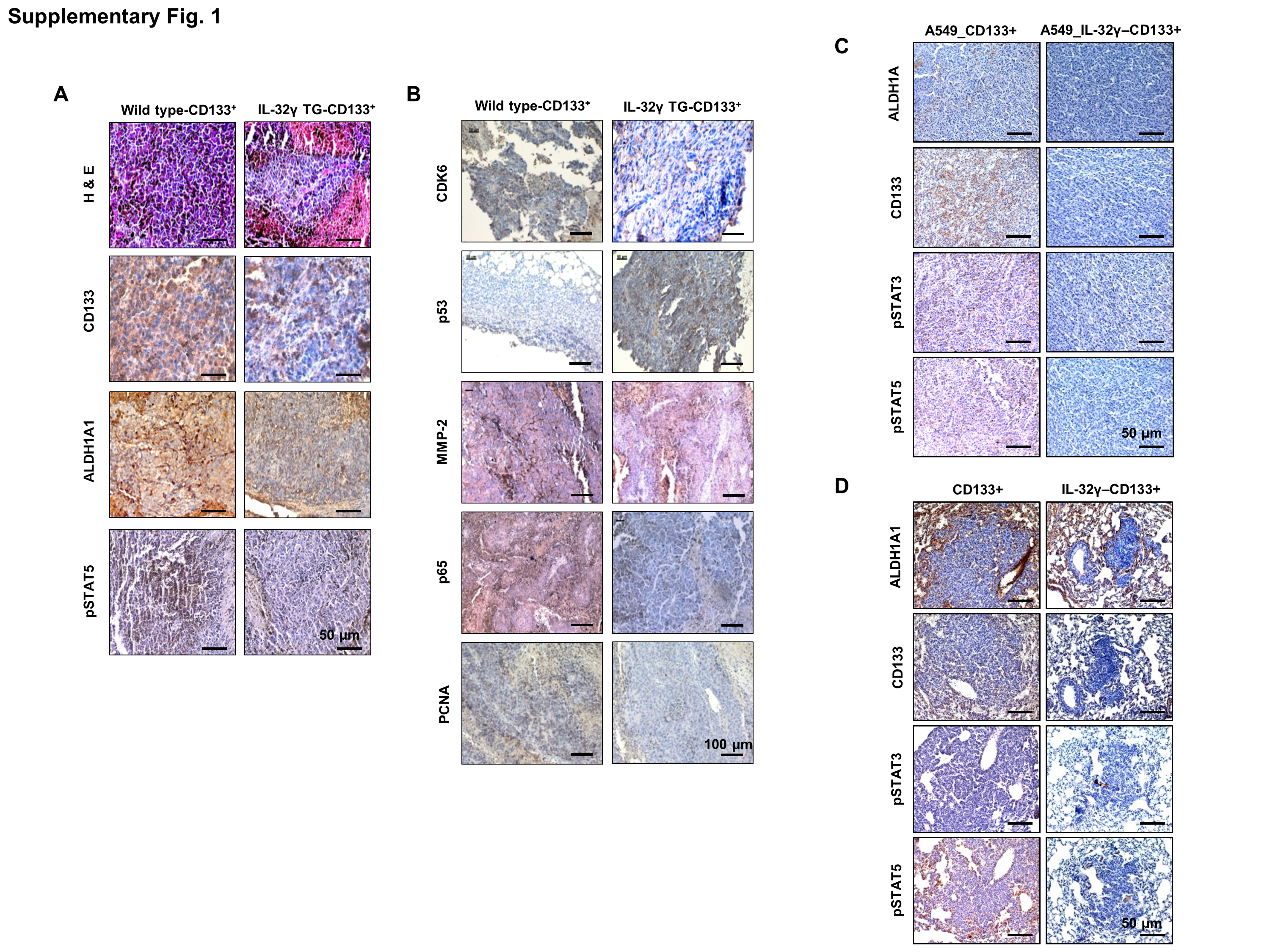

Supplement: Supplementary file 2 — Supplementary figure 1 [file 41419_2019_1737_MOESM2_ESM.tif]

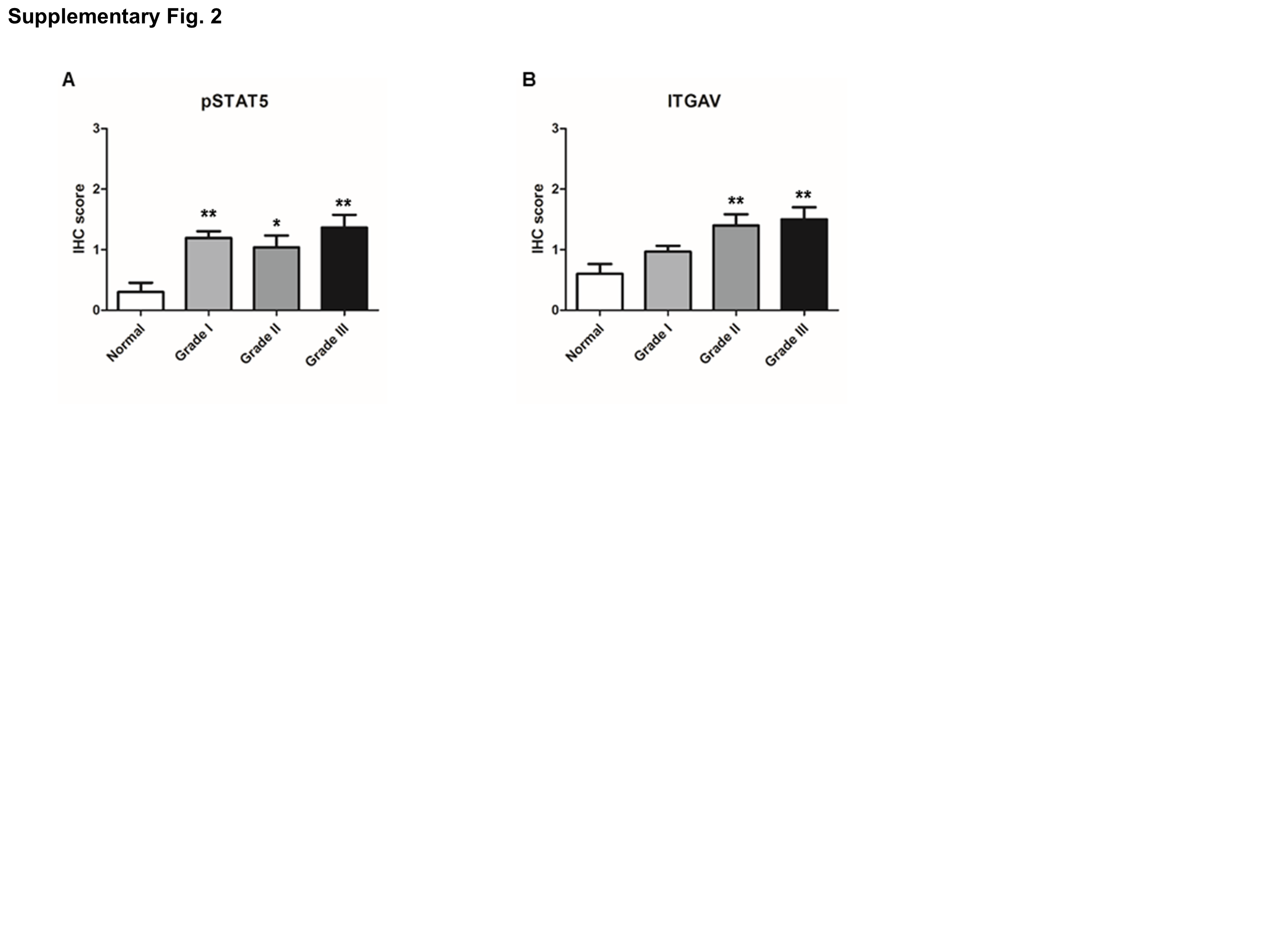

Supplement: Supplementary file 3 — Supplementary figure 2 [file 41419_2019_1737_MOESM3_ESM.tif]

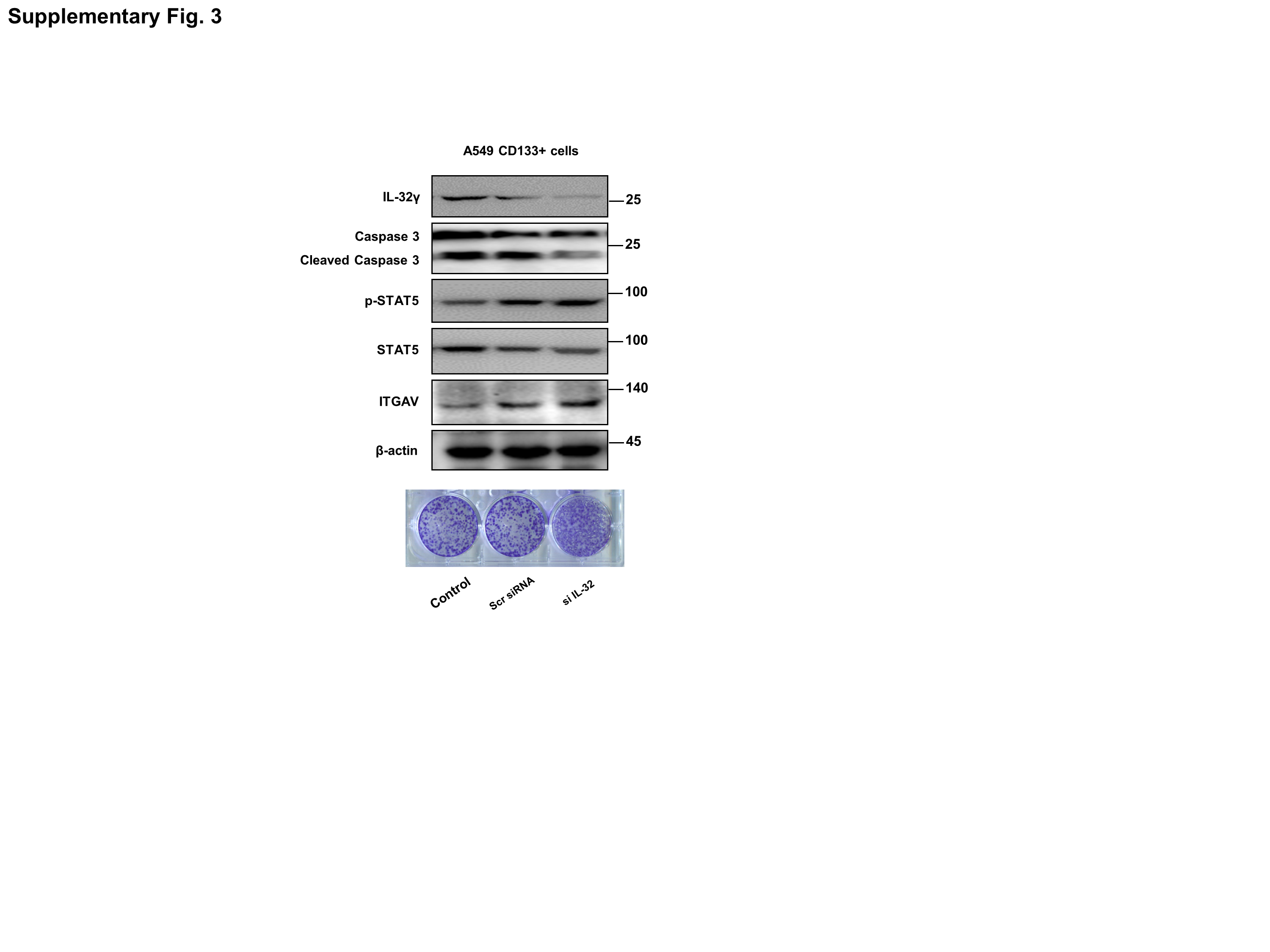

Supplement: Supplementary file 4 — Supplementary figure 3 [file 41419_2019_1737_MOESM4_ESM.tif]
